# Effects of Laser Power Level on Microstructural Properties and Phase Composition of Laser-Clad Fluorapatite/Zirconia Composite Coatings on Ti6Al4V Substrates

**DOI:** 10.3390/ma9050380

**Published:** 2016-05-17

**Authors:** Chi-Sheng Chien, Cheng-Wei Liu, Tsung-Yuan Kuo

**Affiliations:** 1 Department of Orthopaedics, Chimei Foundation Hospital, Tainan 710, Taiwan; jannie.gissing@msa.hinet.net; 2 Department of Electrical Engineering, Southern Taiwan University of Science and Technology, Tainan 710, Taiwan; 3 Department of Mechanical Engineering, Southern Taiwan University of Science and Technology, Tainan 710, Taiwan; ma010115@stust.edu.tw

**Keywords:** fluorapatite, zirconia, laser cladding, laser power, composite coating

## Abstract

Hydroxyapatite (HA) is one of the most commonly used materials for the coating of bioceramic titanium (Ti) alloys. However, HA has poor mechanical properties and a low bonding strength. Accordingly, the present study replaces HA with a composite coating material consisting of fluorapatite (FA) and 20 wt % yttria (3 mol %) stabilized zirconia (ZrO_2_, 3Y-TZP). The FA/ZrO_2_ coatings are deposited on Ti6Al4V substrates using a Nd:YAG laser cladding system with laser powers and travel speeds of 400 W/200 mm/min, 800 W/400 mm/min, and 1200 W/600 mm/min, respectively. The experimental results show that a significant inter-diffusion of the alloying elements occurs between the coating layer (CL) and the transition layer (TL). Consequently, a strong metallurgical bond is formed between them. During the cladding process, the ZrO_2_ is completely decomposed, while the FA is partially decomposed. As a result, the CLs of all the specimens consist mainly of FA, Ca_4_(PO_4_)_2_O (TTCP), CaF_2_, CaZrO_3_, CaTiO_3_ and monoclinic phase ZrO_2_ (m-ZrO_2_), together with a small amount of θ-Al_2_O_3_. As the laser power is increased, CaO, CaCO_3_ and trace amounts of tetragonal phase ZrO_2_ (t-ZrO_2_) also appear. As the laser power increases from 400 to 800 W, the CL hardness also increases as a result of microstructural refinement and densification. However, at the highest laser power of 1200 W, the CL hardness reduces significantly due to the formation of large amounts of relatively soft CaO and CaCO_3_ phase.

## 1. Introduction

Biomedical implants typically comprise a thin bioceramic coating deposited on a titanium (Ti) alloy substrate [1,2,3]. Of the various coating materials available, hydroxyapatite (Ca_10_(PO_4_)_6_(OH)_2_; HA) is one of the most commonly used since it has the same chemical composition and crystallographic structure as the apatite of living bones, and hence promotes early bonding between the implant and surrounding tissue [4,5,6,7]. HA coatings are generally deposited using a plasma spraying technique due to its short operation time, high accumulation rate, low processing cost, and low heat input. However, HA is intrinsically brittle, and hence the as-sprayed HA coatings generally have poor adhesion with the Ti substrate [8]. Moreover, under high temperature plasma spraying, the HA decomposes into impurities and low-crystallinity phases (e.g., tricalcium phosphate (Ca_3_(PO_4_)_2_), tetracalcium phosphate (Ca_4_(PO_4_)_2_O), and calcium oxide (CaO)). These phases cause the coating to delaminate or flake, and can therefore lead to various medical disorders [9]. Furthermore, the HA coating and Ti substrate have very different thermal expansion coefficients. Consequently, significant residual stress is formed at the interface between the coating and the substrate during the spraying process [9]. This residual stress prompts premature coating degradation and can lead to implant failure after long-term imbedding in the human body. As a result, the need for alternative coating materials with improved structural stability under high temperature conditions has emerged as a pressing concern in recent decades.

Fluorapatite (Ca_10_(PO_4_)_6_(F)_2_; FA) has a similar level of biological activity and biocompatibility in the human body as HA. However, FA has better thermal and chemical stability, and therefore reduces the risk of pyrolysis [10,11,12,13]. Furthermore, FA has a slow bio-resorption rate, and thus promotes bone fixing and bone ingrowth [14,15]. As a result, FA has attracted significant interest as a possible substitute for HA in biomedical implant applications [16,17,18]. Many studies have shown that the mechanical properties and biological performance of biomedical coating materials can be enhanced through the addition of secondary phases such as zirconia (ZrO_2_) or alumina (Al_2_O_3_) [19,20,21,22,23,24,25,26]. Among these phases, ZrO_2_ is a particularly attractive choice due to its relatively high mechanical strength and fracture toughness [23,24,25,26].

The excellent mechanical, electrical, thermal and optical properties of zirconia ceramics render them useful for many structural and functional applications [27,28,29,30]. In the biomedical field, yttria-stabilized tetragonal zirconia (Y-TZP) is widely used for dental restoration work due to its high biological safety and good hydrothermal stability [31]. Various studies have shown that 3 mol % yttria (3Y-TZP) provides an ideal stabilizing effect for zirconia [27,32]. However, even though FA is known to be more thermally and chemically stable than HA, the literature contains little information regarding the mechanical and biological properties of FA coatings mixed with ZrO_2_ [4,23,24,31]. Moreover, of those studies which have been performed, the coatings are generally prepared using sintering or plasma spraying techniques. In contrast to such methods, laser beams have high coherence and directionality, and thus have the potential to generate strong metallurgical bonds between the bioceramic coating layer and the substrate [33,34,35,36,37]. Consequently, the present study investigates the microstructural properties and phase composition of composite FA coatings containing 20 wt % yttria (3 mol %) stabilized zirconia (ZrO_2_, 3Y-TZP) deposited on Ti6Al4V substrates using a laser-cladding technique. As in previous studies by the present group [38,39,40], the laser cladding process is performed using an Nd:YAG laser system.

In laser cladding processes, the microstructural properties and phase composition of the coatings are significantly affected by three experimental parameters, namely the specific energy (Es = P/(V × D), P: power; V: travel speed; D: laser beam diameter), the laser power density (LPD = P/(πD^2^/4)) and the interaction time (t = D/V)). In the present study, the cladding process is performed using three different settings of the laser power and travel speed, namely 400 W/200 mm/min, 800 W/400 mm/min, and 1200 W/600 mm/min, respectively. In other words, the laser-power/travel-speed (P/V) ratio is equal to 2:1 in all of the cladding trials. Moreover, the laser spot diameter is equal to 3 mm (approximately) in every case. Consequently, the specific energy is a constant in the present study. By contrast, the laser power density increases as the laser power is increased from 400 W to 1200 W. Finally, the interaction time reduces as the travel speed increases.

## 2. Experiments

**Figure 1:**
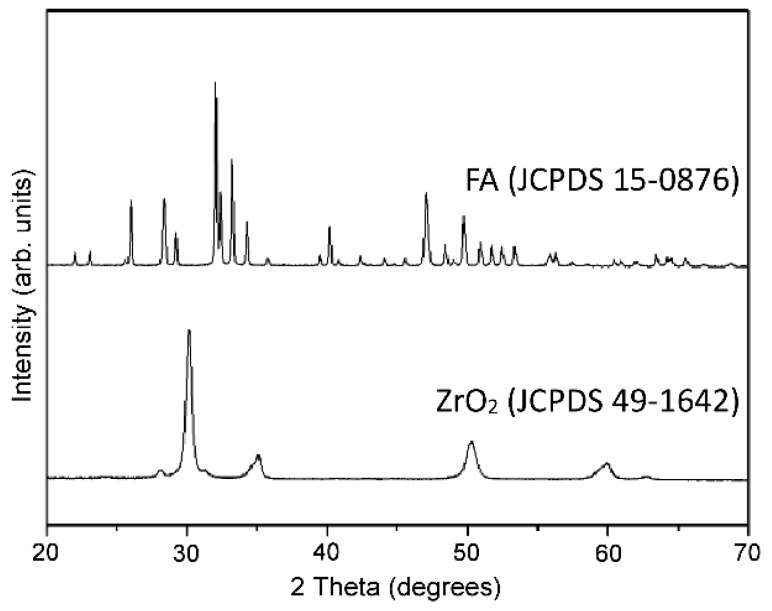
XRD patterns of coating powders: FA and ZrO_2_ (3 mol % yttria (3Y-TZP), partially stabilized with 3 mol % Y_2_O_3_).

**Figure 2:**
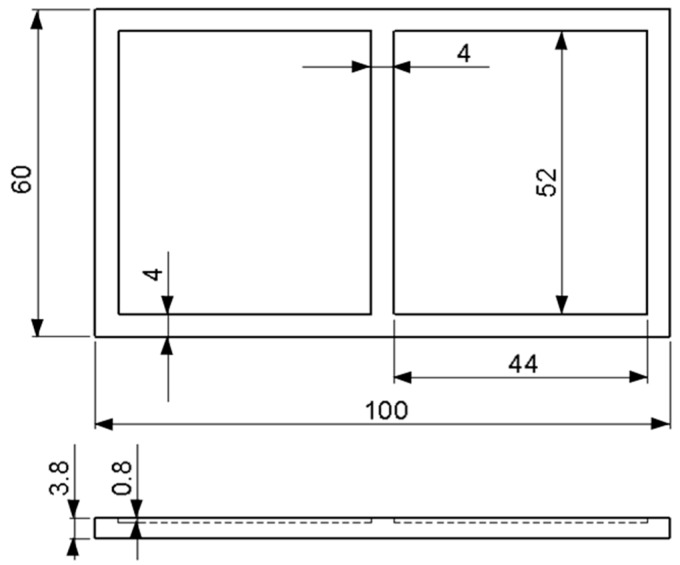
Schematic illustration of Ti6Al4V substrate (unit: mm).

The cladding trials were performed using Ti6Al4V alloy plates with dimensions of 100 mm × 60 mm × 3.8 mm and the chemical composition shown in Table 1. The FA was prepared using Ca_3_(PO_4_)_2_ (*β*-TCP, *β*-tricalcium phosphate) and CaF_2_ (Sigma-Aldrich, St. Louis, MO, USA) powders in accordance with the solid state reaction 3Ca_3_(PO_4_)_2_ + CaF_2_ → Ca_10_(PO_4_)_6_F_2_ [41]. Briefly, the powders were mixed in a stoichiometric molar ratio of 3:1 and milled with ZrO_2_ balls in ethanol for 48 h. After drying, the powder was compacted and heated at 1000 °C for 3 h in air to form solid FA cylinders. The cylinders were then ground into powder and reinforced with 20 wt % commercial ZrO_2_ powder (3 mol % Y_2_O_3_, EE-TEC Inc., Zhongli, Taiwan). Prior to the reinforcement process, the atomic structures of the FA (JCPDS 15-0876) and ZrO_2_ (3Y-TZP, JCPDS 49-1642) powders were examined using XRD (Figure 1). No separate yttria peak was detected (*i.e.*, the XRD patterns of ZrO_2_ (3Y-TZP) and TZP (tetragonal ZrO_2_, t-ZrO_2_) are very similar). The FA/ZrO_2_ powder was mixed with a polyvinyl alcohol binder ((C_2_H_4_O)_n_) in a 3:1 ratio (wt %) and stirred until a slurry-like consistency was obtained. As shown in Figure 2, the Ti6Al4V substrates used in the present study were milled with two slots (each with a size of 52 mm × 44 mm × 0.8 mm). The FA/ZrO_2_ + binder slurry was placed in each slot and the excess quantity removed using a stainless steel scraper. The substrate was then dried in an oven at 100 °C for 30 min under atmospheric conditions. Finally, the specimens were laser clad using an Nd:YAG laser system (ROFIN CW025, 2500 W; Rofin Sinar Technologies Inc., Hamburg, Germany) operating in a continuous-wave mode. For each specimen, the cladding process was limited to a single laser line scan.

**Figure 3:**
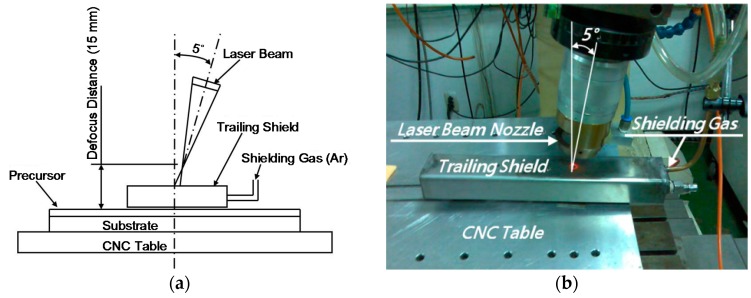
(**a**) Schematic illustration of Nd-YAG laser cladding system which was controlled by the computer numerical control (CNC); and (**b**) photograph of experimental setup.

As stated above, the cladding trials were performed using laser powers (P) and travel speeds (V) of 400 W/200 mm/min, 800 W/400 mm/min, and 1200 W/600 mm/min. The laser spot diameter was equal to approximately 3 mm in every case. Consequently, the specific energy (Es = P/(D × V)) for each coating was equal to 40 J/mm^2^. Furthermore, the laser power densities (LPD = P/(πD^2^/4)) for the three cases were 5.66, 11.32 and 16.98 kW/cm^2^, respectively. Finally, the interaction times (t = D/V) were 0.9, 0.45 and 0.3 s, respectively. The laser beam was guided to the workstation by an optical fiber with a core diameter of 600 µm and a focal length of 120 mm. The cladding process was performed in an Ar-shielded atmosphere (Ar flow rate: 25 L/min) with a 5° laser incident angle and a 15 mm positive defocus length. The experimental setup is shown in Figure 3.

The microstructures of the clad specimens were observed using SEM (JEOL JSM-6390LV, JEOL Ltd., Tokyo, Japan). In addition, the phase compositions were examined using SEM and EDS. Moreover, the various phases were identified using XRD (Cu Kα radiation, Rigaku D/Max Ш.V, Rigaku Ltd., Tokyo, Japan) with a 2θ scanning range of 20°~70° and a scanning rate of 2° min^−1^. Finally, the hardness of the coatings was determined using a micro-Vickers hardness tester under a maximum indentation load of 300 g.

## 3. Results and Discussion

### 3.1. Morphology and Microstructure of Weld Beads

**Figure 4:**
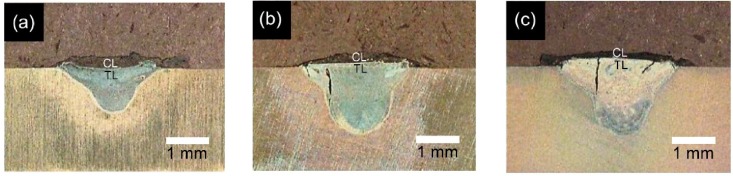
Optical microscope images of weld beads in fluorapatite (FA)/ZrO_2_ laser-clad specimens: (**a**) 400 W, 200 mm/min; (**b**) 800 W, 400 mm/min; and (**c**) 1200 W, 600 mm/min. (Note: CL denotes coating layer and TL denotes transition layer).

Figure 4 presents cross-sectional optical microscope (OM) images of the coating layers (CLs) and transition layers (TLs) of the three laser-clad specimens. As shown, for a constant Es, the depth, width and depth/width ratio (*i.e.*, aspect ratio) of the TL all increase with an increasing laser power (see also Table 2). In addition, cracks are evident within the TLs of the specimens prepared at higher laser powers of 800 W and 1200 W, respectively. The severity of the cracks increases with increasing power.

Given the same laser spot size and thermal diffusivity of the substrate, the size and shape of the weld fusion zone (*i.e.*, weld bead) formed in the laser cladding process depends on the laser power density (LPD). For a low LPD, the weld fusion zone tends to be shallow and bowl-shaped. By contrast, for higher LPDs, the fusion zone is deeper and has a higher aspect ratio [42]. In the present study, even though the LPD increases with an increasing laser power, the specific energy, Es, is fixed. Thus, it seems reasonable to assume that the weld fusion zone should have a similar profile for all three specimens. However, in maintaining a constant Es, the laser travel speed is increased proportionally with increasing laser power. In practice, the laser speed also affects the shape of the weld bead. Thus, as shown in Figure 3, the weld bead profile changes with a changing LPD despite the constant Es in every case.

Previous studies have shown that if the laser cladding parameters are not properly controlled, cracks are readily formed in the coating as a result of the thermal expansion mismatch between the coating and the substrate and relatively low toughness of the coating material [43]. As shown in Figure 4, cracks were not formed in any of the CLs. However, for the specimens prepared using laser powers of 800 W and 1200 W (*i.e.*, a higher travel speed, heating rate and cooling rate), cracking occurred in the TL (see Figure 4b,c). The formation of these cracks is closely related to the residual stress generated during the cladding process [44]. Moreover, the stress increases with an increasing heating and cooling rate. The OM images in Figure 4b,c show that the cracks in the two specimens initiate within the TL, *i.e.*, they do not spread from the CL. It is therefore concluded that the thermal expansion mismatch between the CL and the TL plays no role in prompting crack formation in the TL. In other words, crack initiation is dominated by the thermal stress generated by the rapid cooling rate of the substrate. More specifically, for a higher laser power (a higher laser travel speed), the cooling rate is increased. Consequently, the thermal shock within the TL is enhanced, and hence the crack severity increases. Furthermore, under a higher power level, the weld zone size also increases. As a result, a larger shrinkage stress is induced, and thus crack formation is further enhanced. Notably, however, the OM images in Figure 4 show that crack formation can be controlled through an appropriate setting of the laser power and travel speed parameters.

**Figure 5:**
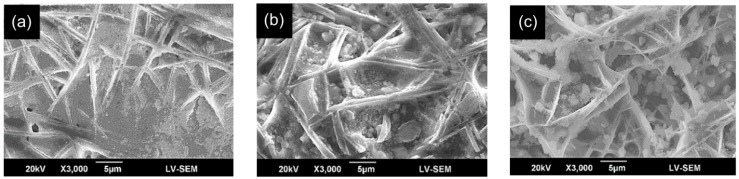
SEM metallographs of CL surface in FA/ZrO_2_ laser-clad specimens: (**a**) 400 W, 200 mm/min; (**b**) 800 W, 400 mm/min; and (**c**) 1200 W, 600 mm/min.

Figure 5 shows the surface microstructures of the CLs in the three laser-clad samples. It is seen that for all three samples, the coating has a fibrous-like morphology. Moreover, as the laser power increases, an increasing number of granular compounds are formed between the fibrous-like structures. The SEM images confirm the absence of microcracks in the CL. The lack of cracks is reasonable since the coefficients of thermal expansion (CTE) of FA and ZrO_2_ (3Y-TZP) (*i.e.*, 9.1 × 10^−6^ K^−1^ [45] and 10~12 × 10^−6^ K^−1^ [46], respectively) are quite close to that of the Ti6Al4V substrate (8.8 × 10^−6^ K^−1^ [47]) in comparison to commonly used bioceramic coatings in the past, HA (15 × 10^−6^ K^−1^ [47]). Hence, only limited differential expansion between the CL and the TL occurs. The addition of ZrO_2_ particles with good mechanical strength and high fracture toughness further enhances the cohesive strength of the CL and suppresses microcrack formation. Therefore, the FA/ZrO_2_-substrate CTE mismatch is less than that for a HA/ZrO_2_-substrate system. Consequently, it can be inferred that the present FA/ZrO_2_ coatings have a better adhesive strength with the Ti6Al4V substrate than HA/ZrO_2_ coatings [8,29].

**Figure 6:**
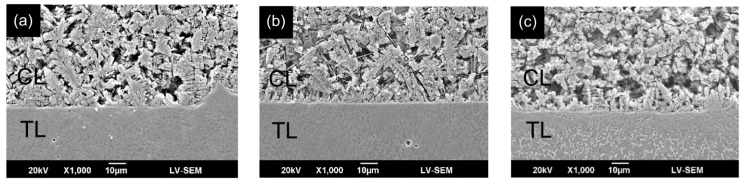
SEM metallographs of CL/TL interface in FA/ZrO_2_ laser-clad specimens: (**a**) 400 W, 200 mm/min; (**b**) 800 W, 400 mm/min; and (**c**) 1200 W, 600 mm/min.

**Figure 7:**
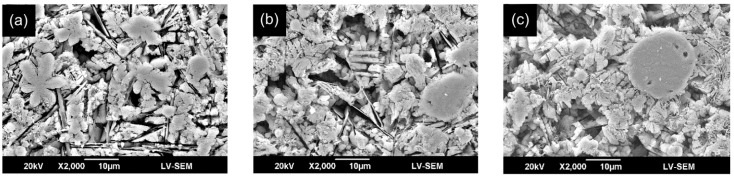
SEM metallographs of mid-section region of cross sectional CL in FA/ZrO_2_ laser-clad specimens: (**a**) 400 W, 200 mm/min; (**b**) 800 W, 400 mm/min; and (**c**) 1200 W, 600 mm/min.

Figure 6 shows the interfacial microstructures of the CL and TL in the three specimens. The EDS analysis results (presented in Section 3.2) show that a significant diffusion of alloying elements occurs between the CL and TL. As a result, a strong metallurgical bond is formed between them [34]. The bonding strength of such an interface is greater than the mechanical bonding strength formed in general coating processes such as plasma spraying, sol-gel, and so on. Figure 7 presents SEM cross-sectional metallographs of the mid-section region of the CL in the various samples. Compared with the CL surface microstructures shown in Figure 5, the quantity of fibrous structures is reduced. However, a large number of spherical and irregularly-shaped particles are observed. For the sample prepared using a low laser power (400 W), the microstructure contains many flower-like structures. However, as the power increases, the flower-like structures are replaced with spherical-like particles. In general, the SEM images presented in Figure 6 and Figure 7 show that the higher cooling rate associated with an increased laser power results in significant microstructural refinement and densification of the CL.

### 3.2. Chemical Composition Analysis of Laser-Clad Coatings

**Figure 8:**
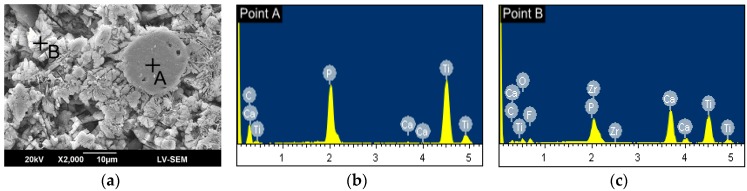
(**a**) SEM metallograph showing microstructure of CL in FA/ZrO_2_ specimen prepared using P = 1200 W and V = 600 mm/min; (**b**) EDS analysis results for particle A in (**a**); and (**c**) EDS analysis results for particle B in (**a**).

Figure 8a presents an SEM image of the CL in the sample prepared using a laser power of 1200 W. Figure 8b,c shows the EDS analysis results for globular particles A and B in Figure 8a, respectively. As shown in Figure 8b, particle A consists mainly of Ti and P. The presence of TiP compounds (Ti phosphides) [48] suggests a partial thermal decomposition of the FA content in the CL during the high-temperature cladding process, accompanied by thermal-induced melting and diffusion of Ti from the substrate. As the laser power increases, the extent of FA decomposition and Ti melting/diffusion also increases, and hence Ti phosphides of a greater size are formed, as shown in Figure 7b,c. It is noted that the present results are consistent with those of Ye *et al.* [48], who showed that a large number of Ti phosphides (Ti_x_P_y_) were formed when sintering Ti/FA (1:1) composite powders under temperatures of 1100 °C or 1200 °C. In general, Ti phosphides can have a wide range of compositions, and it is thus difficult to reliably determine the exact composition of Ti_x_P_y_ by XRD analysis alone [49]. Notably, the EDS results presented in Figure 8c, corresponding to particle B in Figure 8a, show that even under the maximum laser power of 1200 W, the CL matrix still contains a large amount of fluoride. In other words, the potential for FA residue or conversion to fluoride still exists even under high cladding temperatures, as discussed later in Section 3.3.

**Figure 9:**
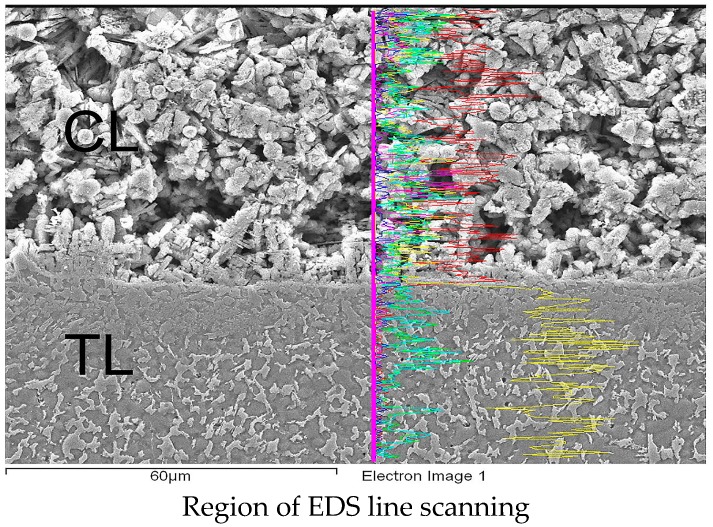

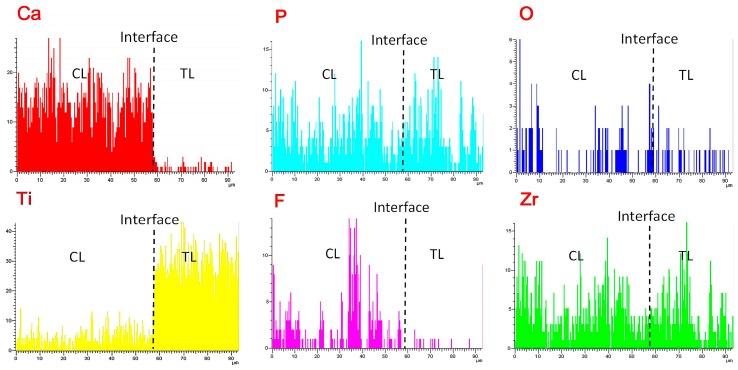
EDS line scan results for Ca, P, O, Ti, F and Zr contents of CL and TL in FA/ZrO_2_ specimen prepared using P = 1200 W and V = 600 mm/min.

Figure 9 presents the EDS line scanning results for the individual alloying elements of the FA/ZrO_2_ cladding layer near the CL/TL interface in the specimen prepared using a laser power of 1200 W. It is seen that the Ca and F elements, *i.e.*, the main decomposition components of FA, are confined almost entirely to the CL. Previous studies have shown that in the thermal decomposition of FA, F is either vaporized as HF gas or forms fluoride [24]. Thus, the F content in the CL layer most probably comes from the FA or fluoride, but requires further XRD analysis for confirmation (see Section 3.3). The P and O ions in the coating material have the ability to diffuse rapidly from the CL to the TL due to their small radii and low activation energy. Nonetheless, as shown in Figure 9, some ions still remain within the CL. It is noted, however, that some of the P ions produced in the FA decomposition process simply vaporize in the high-temperature cladding process [37,38,39,40]. Observing the results presented in Figure 9, it is seen that the distributions of Zr in the CL and TL, respectively, are roughly the same. In other words, given an addition of 20 wt % ZrO_2_ to the FA cladding material, a certain amount of Zr remains within the CL despite the high temperature diffusion effect. Furthermore, the TL retains a very high Ti content; with only a small amount of Ti diffusing to the CL. Overall, the EDS results reveal that while significant diffusion of the alloying elements occurs between the TL and the CL, the alloying elements in the TL are basically similar to the composition of the substrate, while those in the CL are similar to that of the coating material.

### 3.3. XRD Patterns of CL Surface

**Figure 10:**
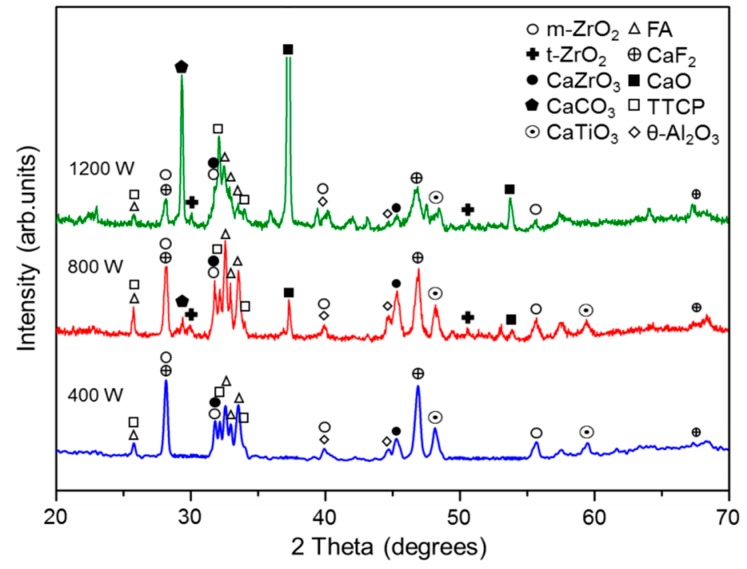
XRD analysis results for CLs of FA/ZrO_2_ specimens prepared using different laser powers.

Figure 10 shows the XRD patterns of the CL surfaces in the three specimens. For the 400 W sample, the CL consists mainly of FA, TTCP (tetracalcium phosphate, Ca_4_(PO_4_)_2_O (JCPDS 25-1137), CaF_2_ (JCPDS 35-0816), CaZrO_3_ (JCPDS 35-0790), CaTiO_3_ (JCPDS 22-0153), m-ZrO_2_ (monoclinic phase ZrO_2_, JCPDS 88-2390) and a small amount of θ-Al_2_O_3_ (JCPDS 23-1009). For the 800 W specimen, the CL additionally contains CaO (JCPDS 37-1497), CaCO_3_ (JCPDS 47-1743) and a trace amount of t-ZrO_2_ (tetragonal phase ZrO_2_, JCPDS 80-0965). For the 1200 W specimen, the CL contains large quantities of CaO, CaCO_3_ and TTCP, but a lesser amount of CaTiO_3_. Furthermore, the quantity of t-ZrO_2_ increases, while that of m-ZrO_2_ and CaZrO_3_ decreases. Notably, the XRD pattern also indicates the presence of trace amounts of several unknown compounds in the CL. In general, the XRD results indicate a greater tendency toward compound phase formation in the CL as the laser power is increased.

The FA powder used in the present study was prepared via a solid state reaction of Ca_3_(PO_4_)_2_ (TCP) and CaF_2_ at 1000 °C for 3 h. However, in the cladding process, the FA reverts to Ca_3_(PO_4_)_2_ and CaF_2_ in accordance with Reaction (1) if the temperature remains sufficiently high for a sufficiently long period of time [23,31]. For example, Nasiri-Tabrizi and Fahami [23] reported that a partial decomposition of FA to Ca_3_(PO_4_)_2_ and CaF_2_ occurs at 900 °C for 1 h in the presence of zirconia. However, at higher temperatures (*i.e.*, greater than 1100 °C), CaF_2_ transforms to CaO through hydrolysis, as shown in Reaction (2) [50]. Thus, the high CaO content in the CL of the present specimen prepared using a high laser power of 1200 W is most likely the result of the thermally-induced hydrolysis of CaF_2_.

(1) Ca_10_(PO_4_)_6_F_2_ → 3Ca_3_(PO_4_)_2_(TCP) + CaF_2_

(2) CaF_2_ + H_2_O_(g)_ → CaO + 2HF_(g)_

In high temperature processes, the decomposition of FA powder without oxide addition can be described by Reaction (3). (Note that Reaction (3) is equivalent to Reaction (1) + Reaction (2)). In other words, in high temperature processes, FA decomposes as TCP and CaO [24,50]. In the present study, ZrO_2_ is also added to the coating. The additional reactions which therefore take place under high density laser power irradiation are described by Reaction (4) below.

(3) Ca_10_(PO_4_)_6_F_2(s)_ + H_2_O_(g)_ → 3Ca_3_(PO_4_)_2(s)_ (TCP) + CaO_(s)_ + 2HF_(g)_

(4) Ca_3_(PO_4_)_2(s)_ (TCP) + 2CaO_(s)_ + ZrO_2(s)_ → Ca_4_(PO_4_)_2_O (TTCP) + CaZrO_3_

Ben and Bouaziz [24] examined the decomposition of FA doped with ZrO_2_, and showed that given sufficient time and temperature, the decomposed TCP and CaO react with the ZrO_2_ to produce TTCP and CaZrO_3_. In addition, many researchers have analyzed the reaction between ZrO_2_ and CaO in accordance with Reaction (5) [4,24,29,51].

(5) CaO_(S)_ + ZrO_2(S)_ → CaZrO_3(S)_

In the XRD patterns in Figure 10, a peak corresponding to the original ZrO_2_ powder (3Y-TZP, XRD pattern similar to t-ZrO_2_) is absent for the 400 W specimen. Moreover, only small quantities of ZrO_2_ are detected in the 800 W and 1200 W samples. In other words, most of the ZrO_2_ particles melt and undergo phase transformation during the laser cladding process. However, FA is still present in all three samples. Thus, it is inferred that even though the original FA powder melts completely during the laser-cladding process, the high thermal stability of the FA powder and the short residence time of the powder at high temperature result in only a partial decomposition of the FA to TTCP and CaO. However, as the laser power increases, the extent of FA decomposition also increases. Thus, for the specimens prepared using higher laser powers of 800 W and 1200 W, respectively, the intensity of the TTCP and CaO peaks in the XRD patterns increases.

Under the high temperatures produced during the laser cladding process, the ZrO_2_ particles reside in a molten state and the diffusion of Ca ions into the ZrO_2_ particles is enhanced. Consequently, CaZrO_3_ is formed during cooling in accordance with Reaction (5) above. Furthermore, CaO is unstable and reacts with CO_2_ to form calcium carbonate (CaCO_3_) in air [31]. As the temperature increases, more of the original FA powder decomposes as CaO, and hence the quantity of CaCO_3_ increases. In addition, Al_2_O_3_ is produced via the reaction between Al atoms diffused from the substrate and the coating material atoms or environmental O atoms. Finally, CaTiO_3_ is produced through a reaction between the decomposed or melted FA and the Ti6Al4V substrate.

For the 400 W sample, the XRD pattern indicates the presence of both m-ZrO_2_ and CaZrO_3_ compounds. For the sample prepared with a higher power of 800 W, the intensity of the m-ZrO_2_ and CaZrO_3_ peaks increases and a trace amount of t-ZrO_2_ emerges. However, for the sample prepared using the highest power of 1200 W, the intensity of the t-ZrO_2_ peak increases, but that of the m-ZrO_2_ and CaZrO_3_ peaks decreases. In other words, the original ZrO_2_ (3Y-TZP) powder melts more completely under high-energy laser irradiation. During the cooling and solidification phase, an allotropic transformation of the ZrO_2_ compound takes place from the cubic phase (c-ZrO_2_ 2370–2680 °C (melting point)) to the tetragonal phase (t-ZrO_2_, 1170 °C–2370 °C), and finally to the monoclinic phase (m-ZrO_2_, room temperature–1170 °C) [32]. For laser cladding at 400 W, the t-ZrO_2_ phase has sufficient time to transform to m-ZrO_2_ due to the lower cooling rate (*i.e.*, the lower travel speed). As a result, the ZrO_2_ exists almost entirely in the monoclinic phase. However, for higher laser powers of 800 W and 1200 W, respectively, insufficient time exists for t-ZrO_2_ transformationto m-ZrO_2_ and consequently the quantity of t-ZrO_2_ increases while that of m-ZrO_2_ decreases. Notably, no c-ZrO_2_ is observed in any of the XRD patterns in Figure 10. This finding suggests that the temperature required for c-ZrO_2_ formation is not maintained for a sufficient length of time during the cooling process, and hence the quantity of c-ZrO_2_ formed is too low to be detected via XRD analysis.

Ramachandra Rao and Kannan [26] and Nagarajan and Rao [51] showed that the CaO released during the sintering of HA/ZrO_2_ at temperatures of 1150 °C and above stabilizes the m-ZrO_2_ via a solid solution reaction, and prompts the formation of t-ZrO_2_. However, with the release of excess CaO through the further decomposition of FA, the solubility of Ca in ZrO_2_ exceeds the maximum solid solution range and hence CaZrO_3_ is formed in preference to t-ZrO_2_ [52,53]. Heimann and Vu [52] showed that when CaO is added to HA/ZrO_2_ composite sintered samples, the surplus CaO is effectively fixed by the ZrO_2_, which acts as a sink for the Ca^2+^ ions. Consequently, either t-ZrO_2_ or CaZrO_3_ is formed. Furthermore, according to the ZrO_2_-CaZrO_3_ phase diagram [54], the formation of CaZrO_3_ depends on the extent of CaO diffusion into ZrO_2_. Hence, a fuller decomposition of FA promotes the production of CaZrO_3_. However, in the present study, although the quantity of CaO increases significantly with an increasing laser power, that of t-ZrO_2_ increases only slightly. Furthermore, the quantity of CaZrO_3_ reduces under the highest laser power of 1200 W. By contrast, the quantity of CaCO_3_ increases significantly with an increasing laser power. This feature suggests that given a sufficiently high laser energy, the tendency of CaO and CO_2_ to react and form CaCO_3_ is higher than that of CaO and ZrO_2_ reacting to form CaZrO_3_. However, further investigation is required to confirm this inference and to clarify the related underlying mechanisms.

### 3.4. Micro-Hardness Evaluation

**Figure 11:**
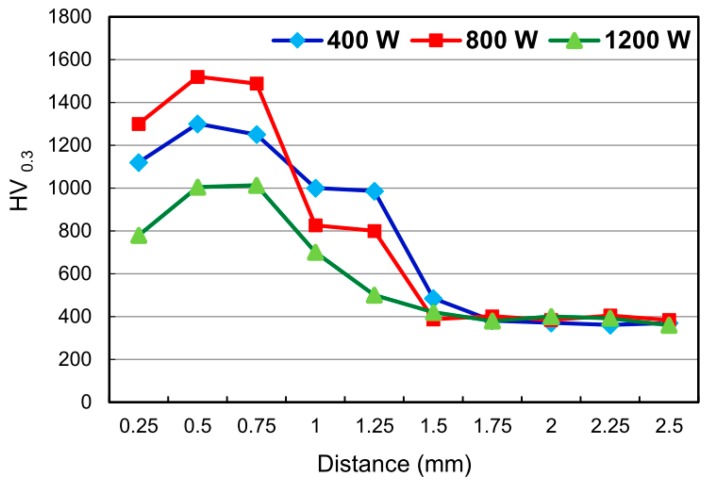
Cross-sectional hardness profiles of FA/ZrO_2_ specimens prepared using different laser powers.

Figure 11 shows the cross-sectional hardness profiles of the various specimens from the CL (thickness approximately 0.2–0.3 mm) through the TL and into the substrate. For all three samples, the TL has a higher hardness than the CL, which in turn has a greater hardness than the substrate. Comparing the CL hardness values of the three samples, it is seen that the hardness increases from 1100 HV_0.3_ to 1300 HV_0.3_ as the laser-cladding power is increased from 400 W to 800 W, but then reduces to around 800 HV_0.3_ as the laser-cladding power is further increased to 1200 W. From inspection, the CL hardness of the three specimens is around 2~3 times higher than that of the Ti6Al4V substrate.

The hardness of laser-clad coatings is related to both their microstructural characteristics (e.g., porosity and density) and their phase constituents [41,43,55]. For the present samples, the CL microstructure exhibits a greater refinement and densification effect as the laser power (travel speed) increases (see Figure 6). Consequently, the hardness increases. The phase constituents of the CL can be ranked in order of decreasing hardness as ZrO_2_ > CaF_2_ > CaO > CaCO_3_ [56]. As shown in the XRD patterns in Figure 10, the sample prepared using a laser power of 1200 W has a high CaO and CaCO_3_ (low hardness) content. Thus, the microstructure-induced hardness enhancement is outweighed by the softening effect of the CaO and CaCO_3_ phases, and consequently a reduction in the CL hardness occurs.

Chien *et al.* [57] showed that the average hardness of the CL formed in the laser cladding of pure FA on Ti6Al4V substrates was equal to 617 HV_0.3_ for a laser power of 740 W and 750 HV_0.3_ for a laser power of 1150 W. It is noted that these hardness values are significantly lower than those obtained in the present study. The laser source and laser cladding parameters are similar in both cases. Hence, it is inferred that the higher CL hardness of the present specimens is due to the addition of ZrO_2_ to the FA matrix. Kim *et al.* [41] prepared sintered FA samples containing 20 and 40 vol % ZrO_2_ powder, respectively, and found that the hardness increased with an increasing ZrO_2_ content. Ouyang *et al.* [43] used a laser-cladding process to deposit yttria partially stabilized ZrO_2_ (7 wt %) ceramic coatings doped with 2.5 wt % TiO_2_ on aluminum alloy substrates. The cladding layer was found to have a hardness of 1415~1575 HV_0.1_. This value is greater than that observed for the present coatings. Kim *et al.* [41] conducted sintering trials using HA and FA powders doped with 20 vol % ZrO_2_. The results showed that the FA-ZrO_2_ composites had a greater hardness (~8 GPa) than the HA-ZrO_2_ samples (~1 GPa). Various studies have attributed the greater hardness of laser-clad ZrO_2_ or ZrO_2_ composite coatings to the absence of porosity and a fine-grained structure [41,43]. However, the present results suggest that the hardness is actually determined by a competition process between the microstructural hardening effects and the phase composition softening effects.

## 4. Conclusions

The present study has deposited composite coatings consisting of fluorapatite (FA) and 20 wt % yttria (3 mol %) stabilized zirconia (ZrO_2_, 3Y-TZP) on Ti6Al4V substrates using a laser cladding process with laser powers and travel speeds of 400 W/200 mm/min, 800 W/400 mm/min, and 1200 W/600 mm/min, respectively. The experimental findings support the following main conclusions.

1. The depth, width and depth/width ratio of the TL increase with an increasing laser power (travel speed). For the 800 W and 1200 W specimens, cracks are formed in the TL due to the greater cooling rate and larger weld zone (*i.e.*, greater shrinkage stress). However, no cracks are formed in the CL due to the addition of ZrO_2_ to the FA powder and the relatively small CTE mismatch between the FA/ZrO_2_ powders and the substrate.
2. A significant diffusion of alloying elements occurs between the CL and the TL. As a result, a good metallurgical bond is formed between them. Overall, the alloying elements of the TL are close to the composition of the substrate, while the alloying elements of the CL are close to the composition of the coating material.
3. The CL of the 400 W specimen consists mainly of FA, TTCP, CaF_2_, CaZrO_3_, CaTiO_3_, m-ZrO_2_ and a small amount of θ-Al_2_O_3_. For the 800 W specimen, the CL also contains CaO, CaCO_3_ and trace amounts of t-ZrO_2_. For the highest laser power of 1200 W, the CaO, CaCO_3_ and TTCP contents of the CL increase significantly. The t-ZrO_2_ content also increases. However, that of m-ZrO_2_ and CaZrO_3_ reduces. In general, the tendency to form composite phases increases as the laser power increases.
4. For all of the specimens, the TL has a greater hardness than the CL. Moreover, the CL hardness is around 2~3 times higher than that of the Ti6Al4V substrate. As the laser power increases from 400 W to 800 W, the CL hardness increases due to a microstructural refinement and densification effect. However, under the highest laser power of 1200 W, the hardness reduces significantly due to the formation of CaO and CaCO_3_ phases with relatively low hardness.

## Figures and Tables

**Table 1:** Chemical composition (wt %) of Ti6Al4V substrates.

Al	V	O	Fe	C	N	H	Ti
6.1	4.24	0.152	0.16	0.017	0.008	0.0006	Balance

**Table 2:** Depth-to-width ratio of TL in FA/ZrO_2_ specimens prepared using different laser powers.

Sample	400 W/200 mm/min	800 W/400 mm/min	1200 W/600 mm/min
Depth (mm)	1.1	1.8	2.0
Width (mm)	2.5	2.9	3.1
Depth/Width Ratio	0.44	0.62	0.65
